# On thermal characterization method of integrated magnetic components

**DOI:** 10.1038/s41598-024-54588-7

**Published:** 2024-02-28

**Authors:** Bechir Mahamat Basma, David Piétroy, Mahamat Issa Boukhari, Zacharia Chahbi, Thomas Blanchet, Jean Pierre Chatelon, Stéphane Capraro, Jean Jacques Rousseau

**Affiliations:** 1 Université Jean Monnet Saint-Etienne, CNRS, Institut d’Optique Graduate School, Laboratoire Hubert Curien UMR 5516, F-42023 Saint-Etienne, France; 2 University of N’Djamena, Farcha Faculty of Exact and Applied Sciences, N’Djamena, Chad; 3 Université de Paris-Saclay, CEA, List, 91120 Palaiseau, France

**Keywords:** Inductors, Fiber Bragg gratings, Resistive sensor, Thermal analysis, Thermal control, Electrical and electronic engineering, Electronics, photonics and device physics

## Abstract

The operating temperature of integrated magnetic components can be critical. Excessively high temperature can significantly modify the properties of components, especially those of magnetic material, such as saturation magnetization and magnetic permeability. This article introduces an experimental characterization method using two different sensors. We compare the results obtained from these sensors. Initially, the method is validated using a “meander component, and subsequently, it is applied to planar spiral inductors, both with and without magnetic material.

## Introduction

In our society, electronic evolutions are driven by the desire of human beings to always get more and more portable technologies: smartphones, computers, connected objects, measurement tools, etc. That is why a special attention is paid to the miniaturization of electronic components in order to reduce both size and weight of final objects^1–3^. Therefore, conductor sections are reduced leading to an increase in current densities then in operating temperatures. This is mainly the case with power components used to convert energy provided by power supply in integrated transformers and DC-DC converters. In such converters, an integrated inductor based on a magnetic core is often the main element of the system. But this component managing the power is often small so its operating temperature is often one of the highest in an electronic circuit. Hence, its electric parameters such as S-parameters, resistance and inductance value, are very sensitive to any temperature change: knowing the operating temperature of the inductor is of the upmost importance.

More generally, due to the heat dissipation in integrated components, the need for thermal analysis of these components during the design phase becomes mandatory^4–6^. Among the main causes of failure of an electronic system, 55% are related to temperature, 20% to vibration, 19% to humidity and 6% to dust^7^. Too high operating temperatures can have several consequences for electronic components as a modification of the electrical properties^8^. This can lead to a circuit which does not operate as the desired function. Secondly, as electronic components are constituted of several materials, the difference in thermal expansion between materials can induce strain inside components. In some cases, cracks can appear, and layers can lift away leading to the component destruction^9^. Then, it is important to estimate real temperature reached inside an electronic component during operation to ensure viability of circuit^10^. Obviously, temperature will increase with the power dissipated by the component. But it will also strongly vary with thermal properties of the component^11^.

Three different heat transfer modes exist. The main one is thermal conduction as occurring inside a solid: thermal energy of both electrons and lattice inside the material is propagating using collisions and vibrations^12,13^. Thermal conduction is modelled using the Fourier Eq. (1)^14^:

1 $${\varPhi }_{cd}=-k.S.\overrightarrow{\nabla }T$$

The conductive heat flux ${\varPhi }_{cd}$ is proportional to the temperature gradient inside the solid and to the surface *S* normal to the heat propagation. This proportionality coefficient named *k* represents the thermal conductivity of the material.

Thermal convection is the second mode. It occurs when a fluid is in contact with a surface having a different temperature. The hot surface heats the fluid in contact, which induces a local modification of density and leads to the fluid movement. The hot fluid moves up and the cold fluid moves down which cools the surface. The convective flux ${\varPhi }_{cv}$ is usually considered as proportional to the temperature difference and to the exchange surface^14^:

2 $${\varPhi }_{cv}=h.S.\Delta T$$

The convection coefficient *h* is in fact very complicated to calculate because it deals with a fluid mechanics problem which depends on the temperature, on the fluid properties and on the geometry of the exchange surface.

Finally, a heat transfer by radiation can be observed between a hot surface and its environment. Heat is converted into electromagnetic energy (photons) and a hot surface act as a light source, reducing the global thermal energy of the object. Radiative flux ${\varPhi }_{rd}$ can be calculated by the following equation:

3 $${\varPhi }_{rd}=\sigma .\varepsilon .S.\left({T}^{4}-{{T}_{amb}}^{4}\right)$$

where *T* is the surface temperature and *T*_*amb*_ the ambient temperature^14^, *σ* is the Planck-Boltzmann constant and *ε* is the emissivity of the material. Radiative flux can be neglected in most of cases when the temperature difference is weak (about few Celsius degrees).

Considering heat transfer, materials and volume will have repercussions on the thermal capacity of the component while the surface will mainly affect radiative and convective fluxes. So, at a constant power dissipated inside an electronic component, the temperature increases as the component size decreases. But the operating temperature problem becomes more critical in the case of integrated circuits such as in power electronic circuits. As known, transformers or DC-DC converters use high currents and powers. Moreover, many of such electronic functions are usually based on planar integrated inductive components. Those components are fabricated using multilayer structures of conductive, magnetic and insulating material. It results in high power components with very small sizes so very low thermal capacity: high temperatures are thus expected.

Temperature simulation can be complex because it deals with a multiphysics problem, which will be even more difficult to model if one considers very high frequency currents e.g., electromagnetic modelling will be necessary. Measurement can be more complicated considering standard temperature probes such as thermocouple or thermos-resistance. These probes are often made of metal inducing a heat conduction leak into the probe and thus an unneglectable error in the measured temperature. Thermal camera is a non-invasive solution, but it is limited in spatial resolution and difficult to calibrate since the emissivity is a complex parameter depending on both the material and its surface roughness^15,16^. The implementation of temperature sensors on embedded components is the new strategy that is becoming widespread because these sensors are easily accessible, occupy small areas and dissipate low energy^17^. In^18^, the authors used optical fibers to measure the temperature variation of a power semiconductor component and evaluate their reliability and lifetime. Their study confirmed that fiber optics proved effective in assessing thermal stress and monitoring the degradation process during acceleration tests, validating its usefulness in this context. Other authors^19^ used FBG to measure the bending temperature of a flexible planar supercapacitor. Their results showed that FBG sensors offer high sensitivity and accurate resolution, making them suitable for monitoring the state of charge of these flexible supercapacitors and opening new possibilities for temperature management. Other works compare characterization methods, including optical fiber and temperature sensitive electrical parameter (TSEP)^20^. In this reference, the authors characterized the junction temperature of an IGBT using both TSEP and fiber optics in presence of silicone gel. Their result indicate that the presence of silicone gel can affect the response of the optical fiber, but the TSEP and optical fiber measurement are consistent when the gel is removed.

In this paper, in order to thermally characterize integrated inductors dedicated to power electronics, we compare two standard temperature measurement methods based on compact sensors: Integrated Resistive Sensor (IRS) and Fiber Bragg Grating (FBG) sensor. Based on electrical measurements, IRS was considered because it can be easily integrated and fabricated during the manufacturing steps of the main inductor track. FBG sensor is based on optical measurements and it was considered because it is a smaller sensor which will both lead to a better spatial resolution and limited thermal disturbances during measurement. Thus, temperature measurement of power integrated inductors is performed. The two presented methods (FBG and IRS), each with their own pros and cons, are non-destructive and can be adapted to components with or without magnetic material. A comparison between them is made. In the first section, we present the tested component and obtained results of generated temperature calculated with COMSOL Multiphysics software. In the second and third sections, we respectively introduce the FBG and IRS measurement methods with the results obtained. In the last section, we compare the two previous methods.

## Tested components

**Figure 1:**
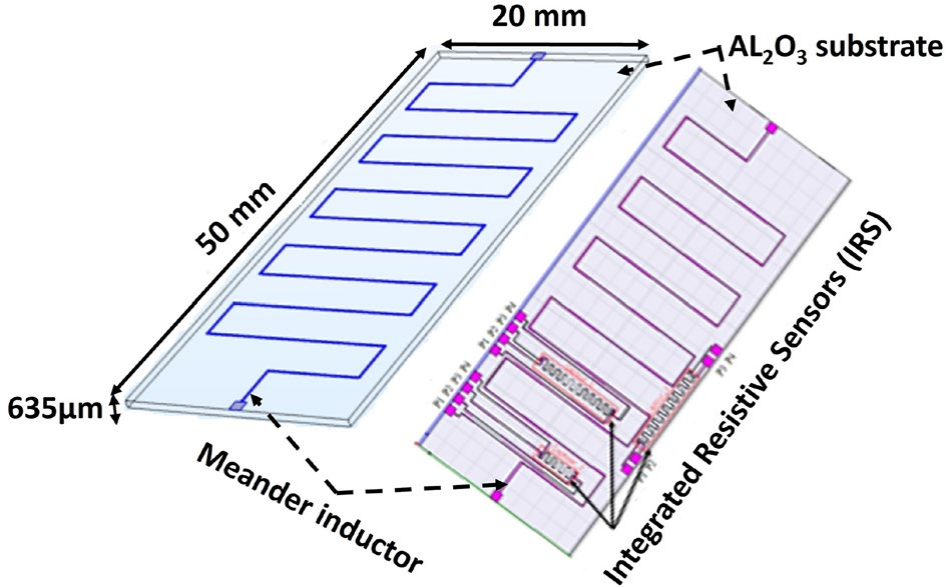
Scheme of the “Meander” inductor with or without IRS.

Two kinds of structures were studied. First, Fig. 1 shows a schematic of the tested planar component, designed in a “meander” configuration. This design was chosen for its ease of integration with a resistive sensor.

**Figure 2:**
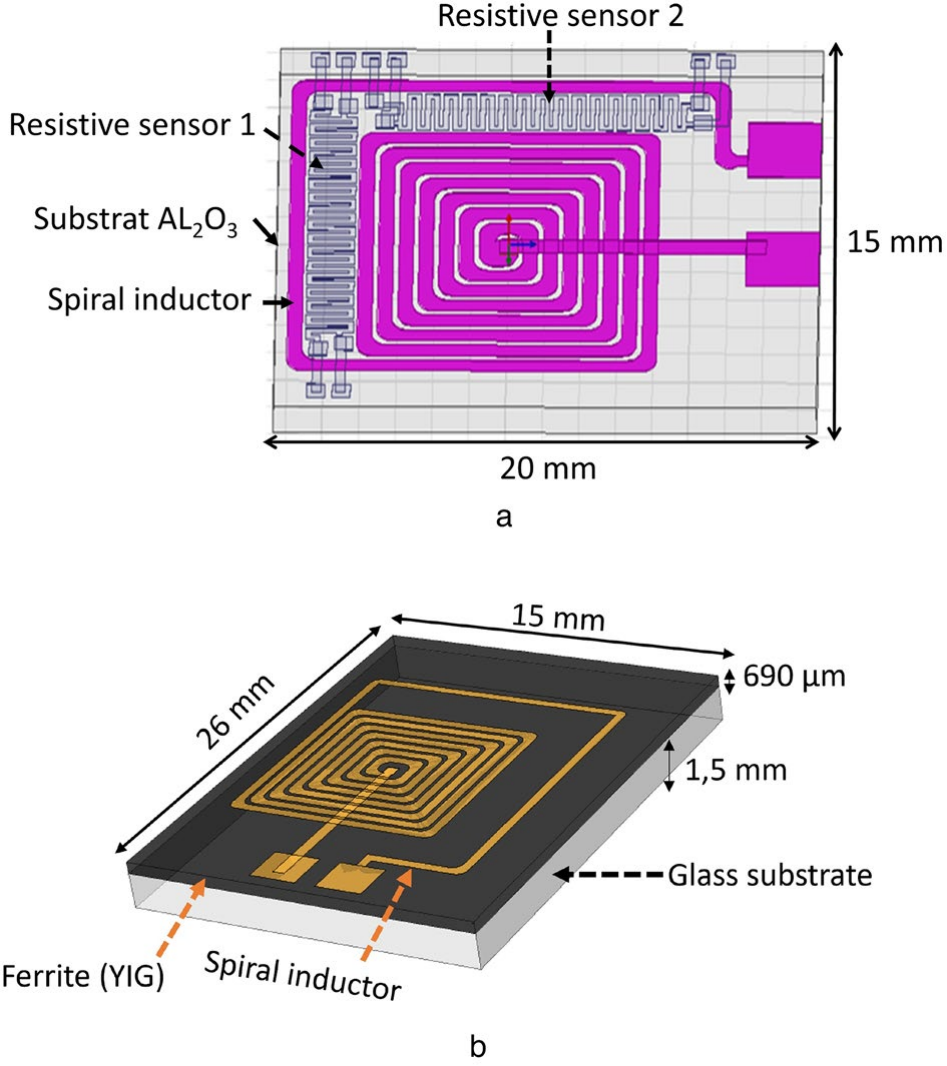
Studied spiral inductors.

The structure consists of a conductor on an Alumina (Al_2_O_3_) substrate, forming a stack of two layers. The conductive track, 5 µm thick, is fabricated on the substrate using standard Micro-Electronics techniques: a layer of copper is deposited by physical vapor deposition (PVD), then patterned using a standard photolithographic process and chemical etching techniques. The conductive path is protected by a finishing layer of gold to prevent from oxidation using an electrolytic deposition. The meander conductor, with a width of 250 µm, reaches a total length of 20 cm and a measured resistance of 8 Ω, determined by impedance measurement and the four-wires method. Secondly, the study focuses on an integrated planar inductor, composed of several thin layers (Fig. 2). This spiral inductor is available in a coreless and a magnetic core version. It is supplied by a DC current. These inductors are also equipped with integrated sensors in the form of small resistors. The conductive part of the component is made of copper deposited on an Alumina (Al_2_O_3_) substrate of 15 × 20 mm^2^ with a thickness of 635 μm (Fig. 2a), or on a ferrite Yttrium Iron Garnet (YIG) substrate of 15 × 26 mm for a thickness of 690 μm (Fig. 2b). The total length, width and thickness of the spiral conductor are 163 mm, 400 μm and 5 μm respectively. These structure presents a classical geometry, but it is complex in terms of realization and heat transfer analysis.

**Figure 3:**
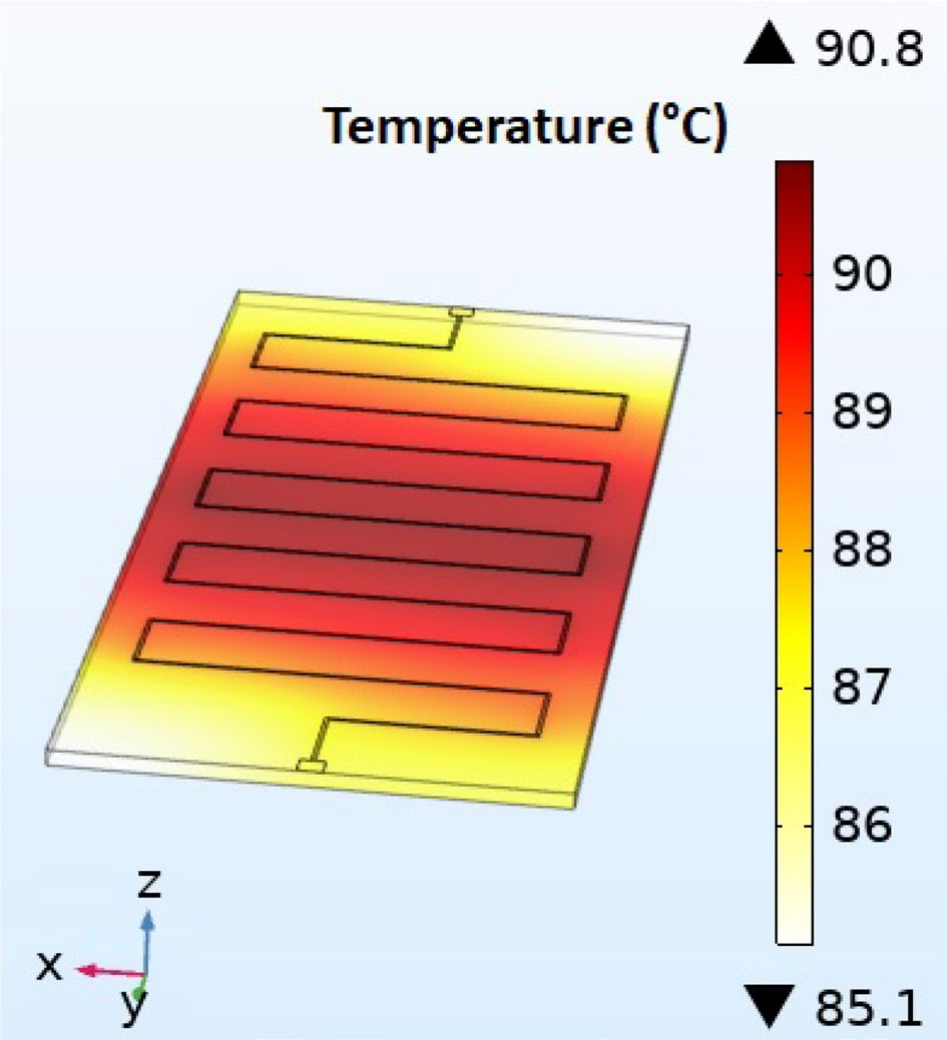
Temperature at the surface of the component for a 0.6 A-current.

All structures have been numerically modelled using COMSOL Multiphysics and its heat transfer module. First, electrical heating based on Joule effect was modelled to quantify localized losses in the volume of the structure. These losses, which represent the power dissipated by the thin conductive layer, are calculated per volume unit and have a direct correlation with heat transfer since it acts as a heat source. The 3D distribution of this heat source is then used to analyze the heat transfers within the component. In this modelling, surface heat flows representing both convection and radiation were introduced at each interface between the component and the air using COMSOL tool. Figure 3 shows a temperature map at the surface of the meander component, corresponding to thermal equilibrium (obtained after prolonged application of power) with a DC input current of 0.6 A. The temperature is almost homogeneous over the entire surface of the component due to the good thermal conductivity of Al_2_O_3_. We can also see that the maximum temperature value is 90 °C.

## Experimental implementation of fiber Bragg grating for temperature sensing

### Measurement principle

**Figure 4:**
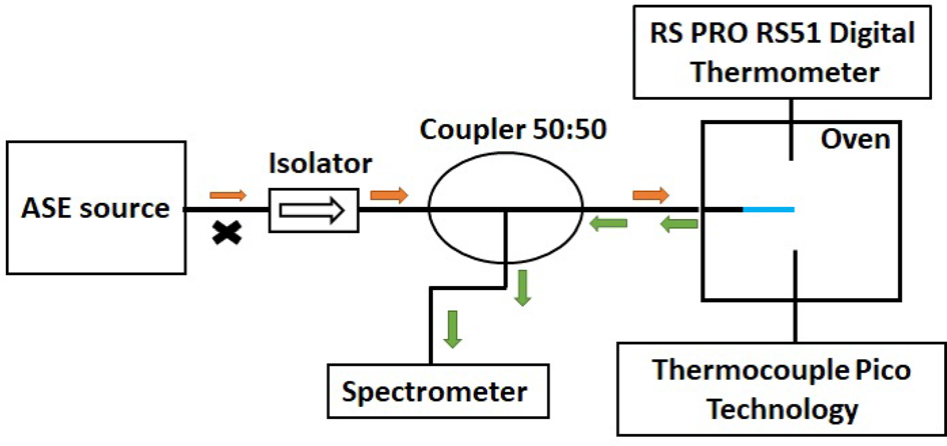
Measurement setup for FBGs in reflection mode.

To perform a temperature/strain measurement with a FBG in reflection mode (Fig. 4), first a broadband source is required. We used an Amplified Spontaneous Emission (ASE) laser source emitting between 1510 and 1590 nm. On its path, light first meets an optical isolator which avoid reflected light to go back and disturb the laser cavity. Then, the light goes through a 50:50 optical coupler which allows 50% of the incident light to be guided toward the grating for its excitation and 50% of the reflected light from the FBG to be guided toward the spectrometer for the measurement. A basic signal processing (background and reference subtraction, curve fitting) is then applied to get the central wavelength of the reflection peak to obtain the Bragg wavelength. In the present work, an I-MON 512 spectrometer is used with a 150 pm resolution. From spectrum, Bragg wavelengths are retrieved using a 50 points-Gaussian fit on each resonance peak ensuring a numerical improvement of the resolution to reach 10 pm.

### FBG inscription

FBGs were photo-inscribed inside a hydrogenated (120 bars–1 week) uncoated SMF-28 single-mode optical fiber from Corning using a UV-CW frequency doubled Argon laser from Coherent emitting at 244 nm at a power of 120 mW. A cylindrical lens is used to focus the laser beam inside the fiber core and a Lloyd mirror is placed between the lens and the fiber to create a periodic interference pattern with an adjustable period by modifying the angle between the mirror and the fiber^21^. With this setup, the laser beam induces optical defects inside the glass leading to an increase in the refractive index. The refractive index of the SMF-28 being about 1.47 at a 1550 nm wavelength, a 530 nm period is necessary to excite a first order resonance close to this wavelength into the Bragg gratings.

**Figure 5:**
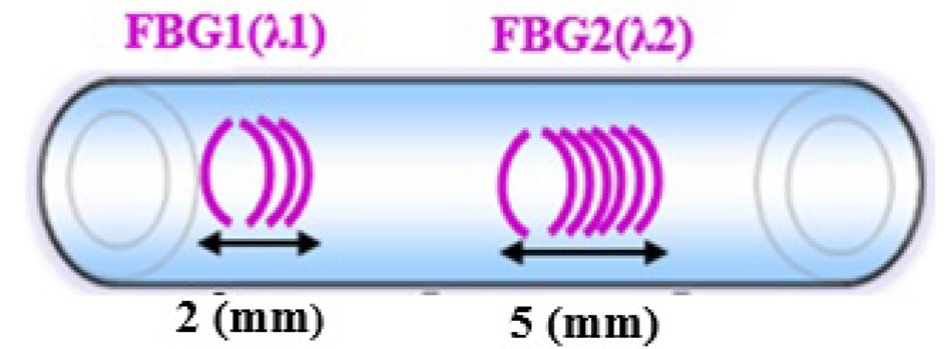
Schematic of the FBG.

**Figure 6:**
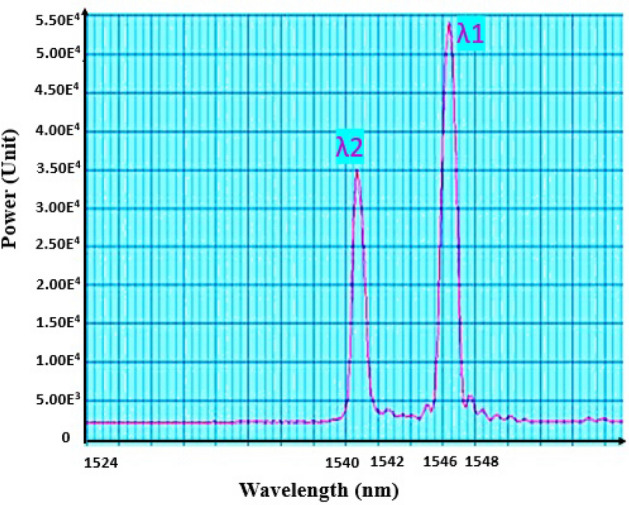
Reflected spectrum of the two FBGs at room temperature (RT).

Two sensors were photo-inscribed at the tip of the fiber to ensure the experiment. Actually, fibers are fragile and the second sensor could be used in the event of breakage during handling, thus avoiding the need to manufacture and calibrate a new FBG from scratch. So, a 2 mm and a 5 mm-long grating were inscribed close to the fiber output to get two temperature sensors (Fig. 5), but only FBG1 was used in this paper. The grating pitch is slightly changed to get a few nanometers variation between the two resonance wavelengths (Fig. 6).

Finally, an annealing is performed in an oven at 120 °C during 10 h to freeze the Bragg gratings. This step removes the remaining hydrogen and stabilizes the grating to operate at lower temperature^22^. An example of reflected signal is given in Fig. 6. At Room Temperature (RT), the 2 mm-long FBG (sensor 1), the closest to the fiber output, exhibits a Bragg resonance at a 1546.1 nm wavelength and the 5 mm-long FBG (sensor 2) exhibits a resonance at 1540.4 nm. The full width at half maximum (FWHM) is about 700 pm.

### FBG calibration and tests

Before using FBG sensors to measure temperature of electronic components, a calibration and a measurement validation must be performed. To calibrate the FBG sensors, the fiber is fixed on the top of an aluminum plate and is then inserted inside an oven. It is important to ensure that the FBGs are stress-free to thermally expand and so avoid strains, which would also contribute to the resonance wavelength change. Two calibrated thermocouples sensors are also used to measure the temperature inside the oven, close to the FBG. The optical fiber is then connected to the ASE light source and the spectrometer. A reference spectrum is acquired at room temperature (RT) and the oven is then heated up to 110 °C. After 30 min waiting for temperature stabilization, a second spectrum at the highest temperature is acquired and the oven is shut down. Temperature inside the oven slowly decreases for several hours during which spectrum and temperature measurements are performed every minute. For temperature measurement, two calibrated thermocouples from RS and Pico Technology were used.

**Figure 7:**
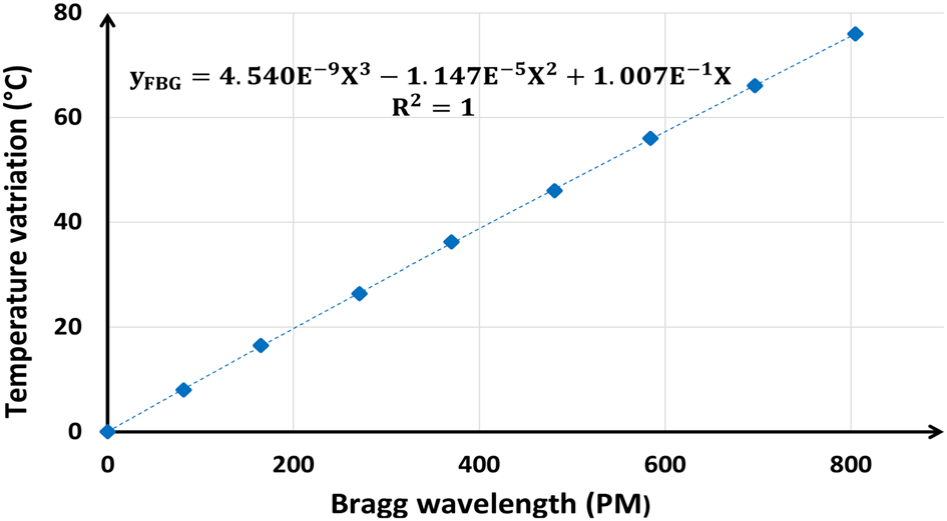
Calibration curves of the FBG.

Considering ambient spectrum and temperature as references, the calibration curve is obtained by tracing the evolution of the temperature difference as a function of the Bragg wavelength shift. Such a curve is presented in Fig. 7. It is to note that the linear approximation of the calibration curve is not suitable for accurate measurements. Therefore, a third order polynomial fit is used.

A second step in the validation of the measurement procedure consists in measuring the response time of the sensor. The FBG sensors are heated on a hot plate then quickly placed in ambient air. Fiber temperature exponentially decreases from hot to room temperature. Spectra are acquired each 10 ms and temperature is then calculated as a function of time. An exponential fit enables the determination of the time constant of the sensor: a 0.35 s time constant was found. It means that the temperature change reaches 90% of the final variation after 0.8 s and 99% after 1.6 s. Hence, FBG is fast enough to thermally characterize the test-component whose time constant was numerically estimated to 32 s in section II.

**Figure 8:**
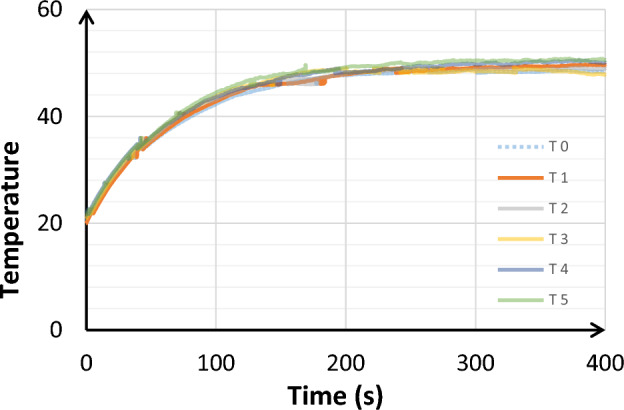
Repeatability tests of FBG sensors.

Finally, the repeatability of the FBG measurements was checked. Gratings were placed in the middle of the test-component which was heated up during 6 min by supplying it with a 0.5 A DC-current. Once the current is off, the FBG cools down in ambient air till it reaches RT. The cycle is repeated 5 times. Temperature measured with FBG is presented on Fig. 8. FBG sensor shows a good repeatability. Nevertheless, a difference was observed between the final temperatures measured.

To conclude this section, let us discuss the measurement error of such a procedure. It is hard to determine the error because there are two main causes: error induced by the calibration and error induced by the measurement. The calibration error is the most difficult to estimate. Main sources are spectrometer resolution, thermocouples calibrations and fit. The main error deals with the fit step. As discussed in section III, for such sensors, an absolute error of ± 1 °C is often considered in the literature. Concerning the procedure error, we showed that the main error deals with the operator. At the end, an error of ± 2 °C seems to be realistic.

### Thermal characterization of the meander component

**Figure 9:**
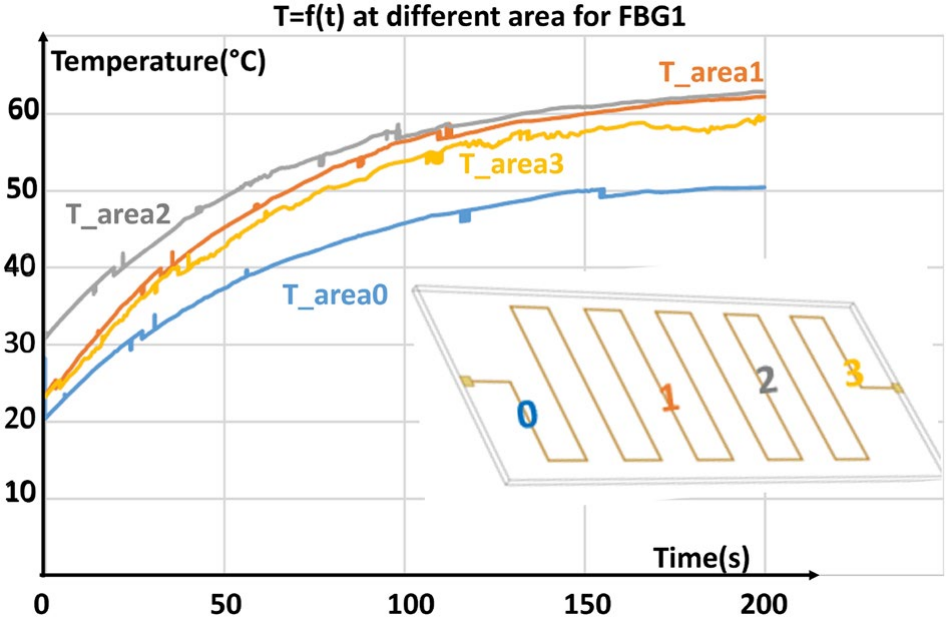
Evolution of temperature along the central axis of the component.

To demonstrate the potential of such sensors for thermal characterization of power electronics components, the test-component was supplied using a 0.5 A DC-current. Then, it is characterized by placing the FBG at different points along the central axis (scheme of Fig. 9).

It is to note that FBG sensors can be precisely placed on the 400 µm-wide copper tracks. Evolution of the temperature along the central axis is presented in Fig. 9. Temperature is quite well homogeneous, but a slight decrease is observed around the edge due to connection wire which foster heat leaks.

## Integrated resistive sensor

### Theory and measurement principle

Resistive sensors are commonly used for temperature measurement. As an example, everyday life sensors called Pt100 use a platinum resistor for temperature measurement. The principle of such sensors is based on the resistivity change with the temperature, leading to a measurable variation in the resistance value of the sensor. A good approximation of the variation of the electric resistivity *ρ* of a material is given by:

4 $$\rho (T)={\rho }_{0}\left[1+\alpha \left(T-{T}_{0}\right)\right]$$

where *ρ* is the resistivity at temperature *T*,* ρ*_*0*_ is the resistivity at the reference temperature *T*_*0*_ and *α* is the thermal coefficient.

Hence, expression (4) can be extended to the temperature dependence of the resistance *R* of a component by using the well-known formula *R* = *ρL/S*, where *L* is the conductor length and *S* its section. According to (4), it is possible to measure the temperature change of a resistive component by measuring its resistance at two different temperatures if its thermal coefficient is known:

5 $$T-{T}_{0}=\frac{R-{R}_{0}}{\alpha {R}_{0}}$$

In the case of integrated resistive temperature sensors, resistance is small, about few ohms. For standard conductive material (*α* about 10^–3^ °C^−1^), the resistance variation considering a 1 °C temperature change is about few milliohms. It is therefore necessary to perform very precise resistance measurements to get a sufficient temperature resolution: a high precision impedancemeter is required. But the measurement setup is also of importance. A standard 2-wires setup would induce measurement errors due to the resistance of connection wires, which would be difficult to consider. A 4-wires method is more suitable for small resistance measurement.

### Temperature measurements using integrated resistive sensors

#### Design—fabrication

Integrated resistive sensors (IRS) were directly integrated on top of the heating component during the fabrication process of the main inductor conductive track. They were placed at specific places (Fig. 1). IRS were designed to exhibit a 2–4 ohms resistance. It deals with a meander copper track 50 µm-wide and 5 µm-thick. IRS are contained in a 2 mm-wide and 10 mm-long area. The total length is about few centimeters. Four pads permit to connect the four wires (high and low currents, high and low voltages) to the sensor. At the end of the fabrication step, the component is stuck onto two PTFE rods to enable a natural convection from both down and up sides.

#### IRS calibration

According to Eq. 5, three measurements are necessary to determine a temperature change using IRS: initial temperature* T*_*0*_, resistance values *R* and *R*_*0*_ at initial temperature. But thermal coefficient *α* has also to be precisely determined to get a better accuracy in temperature measurement. IRS calibration consists in determining this value.

The same method was used as for FBG calibration. The component is heated inside an oven while temperature is directly measured using the same calibrated thermocouples used in the FBG sensor calibration. Then the resistance is measured as a function of component temperature. But the 4-wires device cannot be inserted into the oven, thus a standard 2-wires method using a high precision Keithley 72-7740 ohmmeter was used to directly measure the whole inductor resistance. At ambient temperature, the resistance of the large connection wires was measured to correct component resistance measurements from this systematic error.

**Figure 10:**
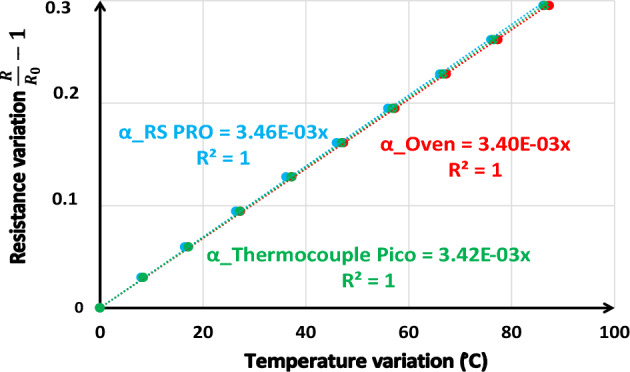
Determination of the thermal coefficient of the copper layer.

Figure 10 presents the evolution of an image of the resistance (*R*/*R*_*0*_-1) upon the temperature. These results are obtained from developed calibration method using the three sensors. According to Eq. 5, *R*/*R*_*0*_-1 is proportional to *ΔT*: a linear regression on the curve of Fig. 10 gives the *α*-value. The thermal coefficient is calculated to be 3.43.10^–3^ °C^-1^.

The accuracy of temperature measurement of these sensors depends on the configuration used. Considering the parameters of Eq. 5 and the impedancemeter measurement errors, we find an accuracy of 2 °C.

## Comparison between FBG and IRS sensors for temperature measurement

### Temporal evolution of the temperature

#### Meander structure

**Figure 11:**
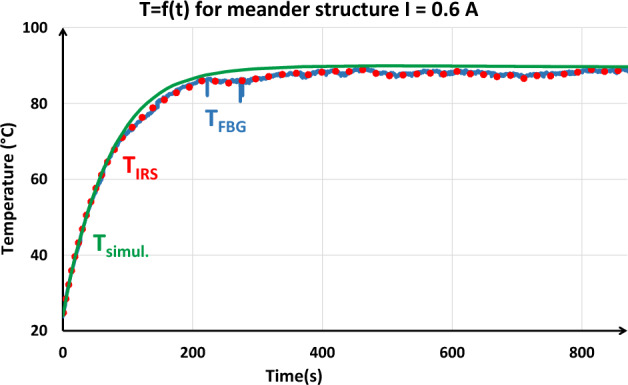
Time evolution of the temperature of the component at the IRS position for a 0.6 A DC-current supply.

In this paragraph, we present the temperature evolution as a function of the time for the meander component supplied by a 0.6 A DC-current. The used sensors are both FBG and IRS sensors. FBG is placed on top of the IRS sensor in order to get a good comparison. Results are given in Fig. 11. Compared to the results from modelling (sec. II, Fig. 3), we find an exponential (IRS and FBG) increase with a time constant of 69.5 s which is close to the 70 s obtained by numerical modelling. Concerning the temperature at thermal equilibrium, modelling and measurement are comparable. As shown in the Fig. 11, the three temperature curves show a good agreement between the measurements of the sensors (IRS, FBG) and simulation. The final temperatures are around 90 °C. Time response of both kind of sensors are good.

In a second time, the thermal influence of each sensor is investigated. First, the component is heated up to 80 °C and the IRS resistance is measured. Once thermal balance is reached, the FBG sensor is placed on top of the IRS and the resistance is measured. The operation is repeated several times. As a conclusion, FBG sensor does not induce any visible resistance change of the IRS sensors. This means that temperature remains unchanged and hence that the FBG sensor does not disturb the thermal behavior of the component. Then the reverse investigation is led: probes are alternatively connected and disconnected from the component. For each test, the Bragg wavelength is measured, and no variation was observed for this large sample.

#### *Spiral inductor on Al*_*2*_*O*_*3*_

Influence of the 4 probes of the LCR_meter on temperature measurement

The aim of the study was to analyze the impact of the probes of LCR meter on the accuracy of temperature measurements in a spiral inductor. Experiments were conducted using an FBG sensor at two levels of current (low 440 mA and medium 714 mA) in two configurations: without contact between the LCR meter’s probes and the resistive sensor, used as a reference, and with contact, to observe the influence of this interaction on the accuracy of the measurements.

**Figure 12:**
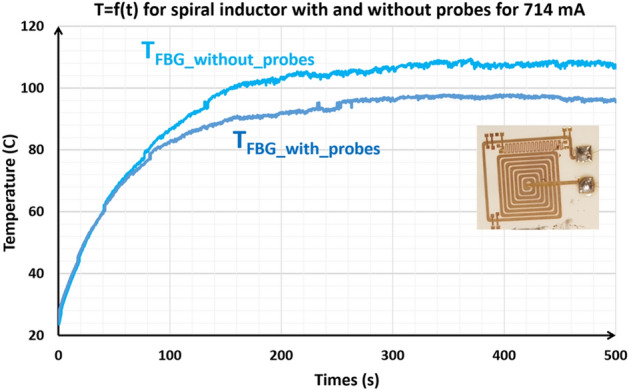
Evolution of the temperature of the spiral inductor with and without probes at current 714 mA.

Figure 12 illustrates the temperature curves for the case where the current applied is 714 mA. The purpose of this comparison is to understand the effect of the thermal shunt produced by the probes on the temperature measurement provided by the resistive sensor when using the spiral inductor. Based on the measurements taken, it is clear that the temperatures recorded without the use of the four probes of the LCR meter are significantly higher, with a difference of 5°Cat a low current of 440 mA and 10 °C at a higher current (714 mA) (Fig. 12). These observations show that the four probes of the LCR meter induce a significant thermal shunt effect. It’s therefore essential to take this effect into account in simulation or to carry out measurements without using the resistive sensor to ensure the accuracy of data.

Comparison of temperature measurements of the spiral inductor obtained by the IRS and FBG sensors at different currents are illustrated in the figure below.

**Figure 13:**
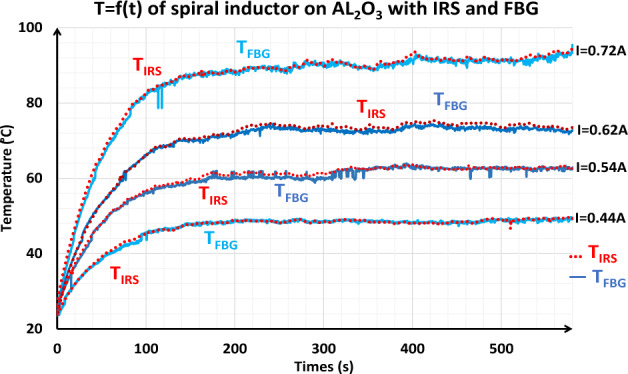
Variation of temperature over time of the inductor on AL_2_O_3_.

Figure 13 shows very good agreement between the FBG and IRS measurements at each given current value. However, the measurements provided by the FBG show some fluctuations at higher currents.

##### Temperature as a function of the dissipated power of the spiral inductor

**Figure 14:**
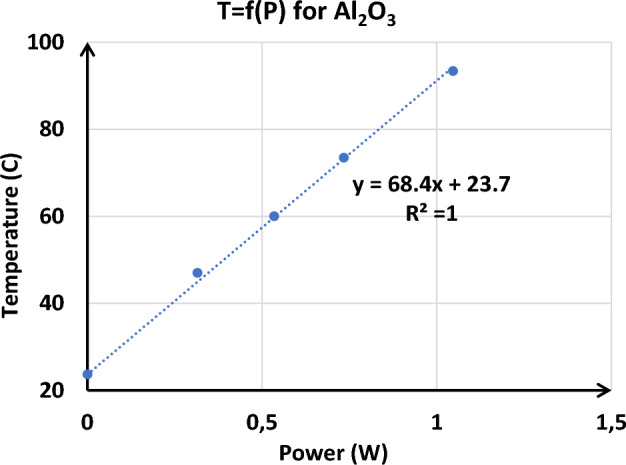
Temperature as a function of dissipated power spiral inductor on Al_2_O_3_.

Figure 14 illustrates the relationship between temperature and dissipated power. It clearly shows that the temperature difference exhibits a linear dependence on the amount of thermal power dissipated. This correlation indicates that as power dissipation increases, temperature rises proportionately, confirming the direct impact of power dissipation on thermal behavior.

##### Comparison between FBG, IRS and simulation for spiral inductor on Al_2_O_3_ (shown on Fig. 2a)

**Figure 15:**
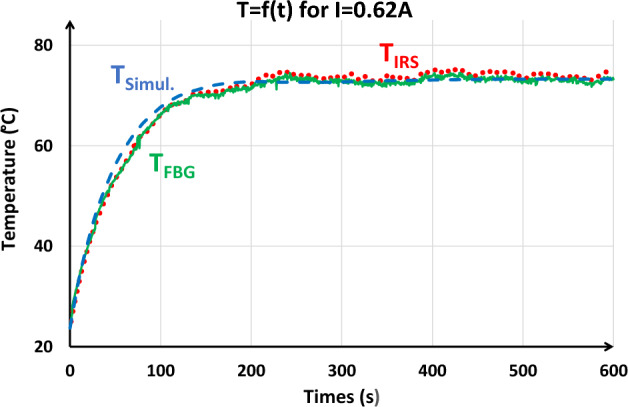
Time evolution of the temperature of the spiral inductor on AL_2_O_3_ with different sensors and simulation for a 0.62 A DC current supply.

Figure 15 presents the temperature evolution of the component from the measurement and the numerical modelling using the two sensors. A good agreement between measurements and simulation is observed. The final temperature measured by the IRS is slightly higher (74.2 °C) than the others (${T}_{FBG}=73.2$ °C and ${T}_{simul.}=73.3$ °C). The relative measurement error is 1.7% for IRS and 0.5% for FBG. The thermal time constant is also measured at 56.8 s in measurements and 56 s in simulation.

#### *Spiral inductor on ferrite (shown on *Fig. 2b*)*

**Figure 16:**
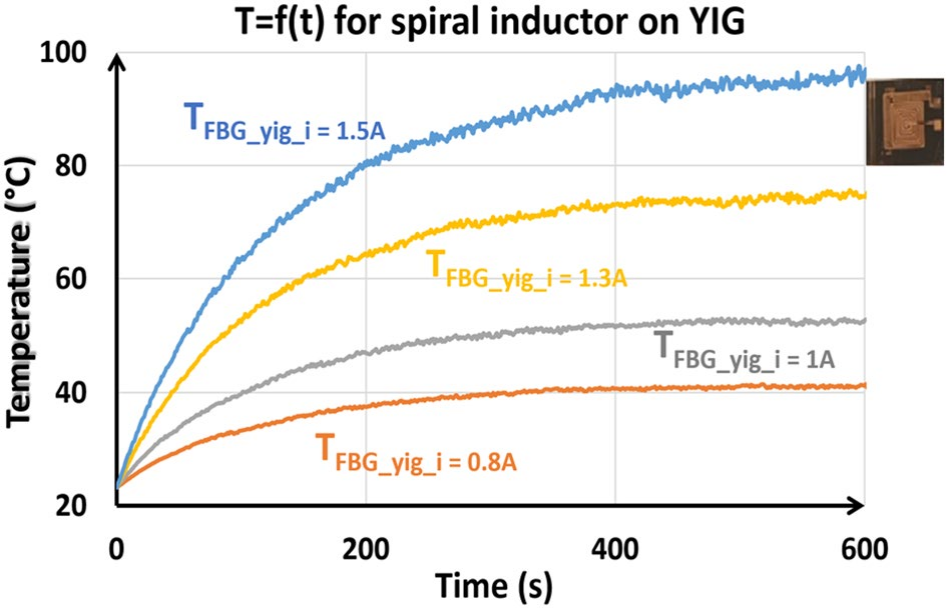
Variation of temperature at different current of the spiral inductor on Yttrium Iron Garnet (YIG).

**Figure 17:**
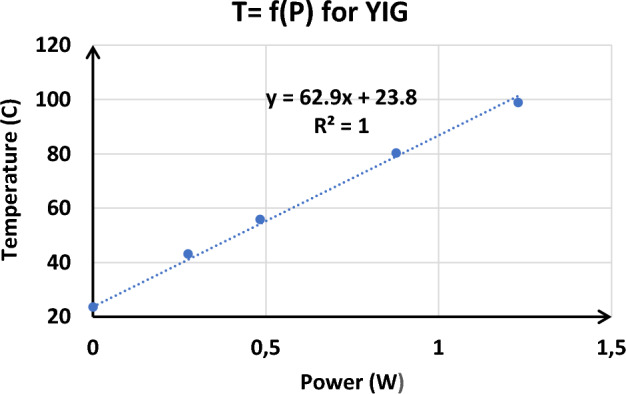
Temperature as a function of dissipated power of spiral inductor Yttrium Iron Garnet (YIG).

Fig.16 shows examples of time evolution of the temperature measured using the FBG for heating DC currents of 0.8, 1, 1.3, and 1.5 A. Temperature reaches 100 °C for currents as low as 1.5A. Figure 17 highlights a linear relationship between temperature and power dissipation in a spiral inductor with magnetic material, demonstrating that an increase in power dissipation leads proportionally to an increase in temperature, thus underlining the direct influence of power dissipation on thermal properties.

### Pros and cons

FBGs are very user-friendly in both fabrication and use. As shown in Fig. 9, they enable the possibility to perform a temperature cartography of the component with a better spatial resolution than fixed IRS sensors. Their temperature measurement accuracy is also as good as the accuracy of IRS sensors (2 °C). They exhibit an acceptable time response compared to quasi-instantaneous IRS sensors: a 0.35 s thermal time constant was measured. As expected, both sensors have a good repeatability.

FBG also exhibit a negligible influence on thermal behavior of the component while IRS sensors thermal disturbance induces an error in temperature measurement increasing with smaller size components.

IRS sensors are closer to the surface than FBG sensors which seems better for temperature measurements. Thus, temperature does not exhibit a significative change at the FBG location (up to 65 µm from the surface). But IRS sensors can be integrated inside a component to measure the inside temperature, which is much more difficult to achieve with a FBG sensor.

Concerning experimental implementation, one FBG sensor (one calibration) can be used thousand times while one IRS sensor (one calibration) only characterizes one component at one point. FBG sensors can also be used far from the light source and spectrometer, even from kilometers if necessary, which makes them easy to install and implement on an industrial manufacturing line. On the contrary, IRS sensors need to keep close to the impedancemeter as connection wires are calibrated for the system device/probes.

## Conclusion

In the first part of this article, two means of thermal characterization of integrated magnetic components were described and compared.

- The use of an integrated resistive sensor (IRS) produced at the same time as the magnetic component without modifying it is well suited, but precautions must be taken to avoid thermal leaks during measurement.
- FBGs are user-friendly and can measure the temperature map of different components with higher spatial resolution. But they are more complicated to make and must be handled with care.

In a second part we implemented and compared these 2 methods on 3 different components: meander spiral, spiral on Al_2_O_3_ and spiral on ferrite.

The experimental demonstration shows that, in both cases, the measurements are in good agreement with the numerically modeled temperature value.

## Data Availability

The data supporting the results of this study are available in the article. Additional information files (data tables and graphs) are available on request from the corresponding author.
